# Social overcrowding impacts gut microbiota, promoting stress, inflammation, and dysglycemia

**DOI:** 10.1080/19490976.2021.2000275

**Published:** 2021-12-02

**Authors:** Clara Delaroque, Mélissa Chervy, Andrew T. Gewirtz, Benoit Chassaing

**Affiliations:** a Inserm U1016, Team “Mucosal Microbiota in Chronic Inflammatory Diseases”, Cnrs Umr 8104, Université de Paris, Paris, France; b Université Clermont Auvergne, Inserm U1071; Usc-inrae 2018, Microbes, Intestin, Inflammation Et Susceptibilité de l’Hôte (M2ish), 28 Place Henri Dunant, Clermont-Ferrand, France; c Center for Inflammation, Immunity and Infection, Institute for Biomedical Sciences, Georgia State University, Georgia California, USA

**Keywords:** Social stress, microbiota, inflammation, metabolic deregulations

## Abstract

An array of chronic inflammatory diseases, including metabolic diseases such as obesity and diabetes, are thought to be promoted by disturbance of the intestinal microbiota. Such diseases disproportionately impact low-income communities, which are frequently afflicted by chronic stress and increased density housing. Hence, we hypothesized that overcrowded housing might promote stress, microbiota dysbiosis, inflammation, and, consequently, metabolic diseases. We tested this hypothesis in a tractable murine model of social overcrowding (SOC), in which mice were housed at twice normal density. SOC moderately impacted behavior in some widely used assays (Open Field, Elevated Plus Maze and Light/Dark tests) and resulted in a stark increase in corticosterone levels. Such indices of stress were associated with mild chronic gut inflammation, hyperglycemia, elevations in colonic cytokines, and alterations in gut microbiota composition. All of these consequences of SOC were eliminated by broad spectrum antibiotics, while some (inflammation and hyperglycemia) were transmitted by microbiota transplantation from SOC mice to germfree mice housed at normal density. Altogether, these results suggest a central role for intestinal microbiota in driving stress, inflammation, and chronic diseases that are promoted by overcrowded housing.

## Introduction

Humanity is increasingly afflicted by an array of chronic inflammatory diseases, including obesity and type 2 diabetes, which are not viewed as infectious diseases per se but yet are associated with, and thought to be promoted by, alterations in gut microbiota^1^^,2^. These diseases disproportionately impact low-income communities^3^, a health disparity likely driven, in part, by differential access to education, healthcare, and high-quality foods. More generally, those residing in low-income communities can be afflicted by chronic stress, which can be physical and/or psychological in nature. Chronic stress is a central factor in diseases characterized by histopathologically evident inflammation such as Inflammatory Bowel Disease (IBD)^4,5^, as well as diseases associated with low-grade inflammation such as irritable bowel syndrome (IBS)^6^ and metabolic syndrome. Incidence of these chronic disorders have increased in recent decades^7,8^, perhaps concomitant with increased stress exposure. More generally, while genetic predisposition has a strong influence on development of chronic diseases, chronic stress can be viewed as an environmental (i.e. nongenetic) factor that might have contributed to increased incidence of these diseases.

Intercommunication between the gut and the brain, referred to as the gut-brain-axis, can occur through the vagus nerve, immune mediators, and neuroendocrine pathways, all of which can influence, and be influenced by, intestinal microbiota^9^. Indeed, recent studies support a role for microbiota in chronic diseases associated with stress and anxiety^10,11^. For example, microbiota removal through use of germfree mice or antibiotic treatment is often sufficient to abrogate intestinal inflammation in mouse models of these disorders^12,13^, while fecal microbiota transplantation demonstrates that select alterations in microbiota can be sufficient to promote chronic intestinal inflammation^14^. Moreover, gut microbiota can impact the brain and behavior, evidenced by the observation that germfree mice lacking microbiota display lower anxiety-like behavior compared to conventional mice^15^, while fecal microbiota transplantation from chronically stressed mice is sufficient to transfer anxiety-like and depression-like behavior in recipient mice^16^. In addition, microbiota transfer from BALB/c mice, which are inherently highly anxious, into germfree NIH Swiss mice (a less-anxious mouse strain) resulted in an increase in anxiety-like behaviors in the recipient mice, whereas microbiota transfer from NIH Swiss mice into germfree BALB/c reduced anxiety^17^. Previous studies also demonstrated that single bacterial species could be sufficient to modulate behavior. As an example, restoring intestinal *Lactobacillus* levels in chronically stressed mice was found to be sufficient to improve stress-associated behavior^18^ and normalize corticosterone levels^19^

One cause of chronic stress in low-income communities is high density housing^20^, which can be envisaged to cause physical and/or psychological alterations/adaptations. Indeed, social overcrowding (SOC), which can be modeled in mice by increasing number of animals in a confined cage, induces anxiety-like behaviors and promotes adiposity without any change in body weight^21^. Analogously, we herein report that SOC led to increased levels of major stress hormone, corticosterone, as well as circulating blood glucose. Importantly, such changes were accompanied by alterations in gut microbiota composition and low-grade intestinal inflammation. Such SOC-induced changes were fully abrogated by broad-spectrum antibiotic-treatment and partly recapitulated by fecal microbiota transplantation, thus supporting a central role played by the intestinal microbiota in gut-brain interactions that drive chronic inflammatory diseases.

## Results

### Social overcrowding-induced stress and altered metabolism

**Figure 1.**
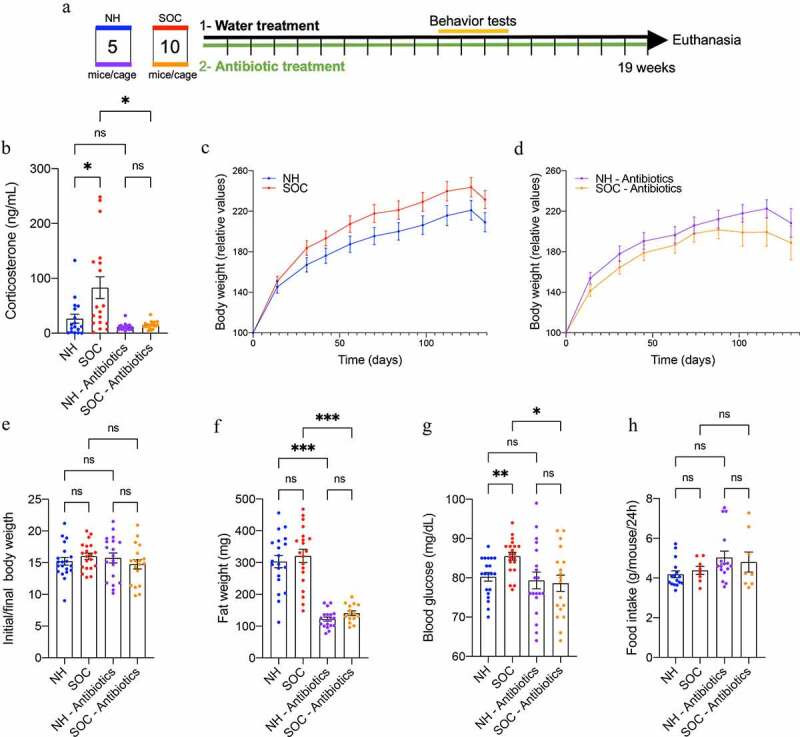
**SOC-induced stress increases molecular markers of stress and fasting blood glucose levels in a microbiota dependent manner**. Mice were subjected to SOC or normal housing for 19 weeks with or without antibiotic treatment, as presented in (a). Corticosterone serum level was analyzed by ELISA (b). Body weight over time of untreated mice (c) and antibiotic treated mice (d); initial body weight to final body weight ratio (e). Epididymal white adipose tissue weight (f); 15 hr fasting blood glucose level after 17 weeks of SOC housing (g), and food intake (h) after 19 weeks of SOC housing. Data are the means ± SEM and points represent individual mice (N = 20). Significance was determined using Kruskal-Wallis corrected for multiple comparisons with a Dunn’s test, or Brown-Forsythe and Welch ANOVA corrected for multiple comparisons with a Dunnett test, or one-way ANOVA corrected for multiple comparisons with a Bonferroni test or a two-way ANOVA corrected for multiple comparisons with a Bonferroni test (*p ≤ 0,05; ***p ≤ 0,001; n.s. indicates nonsignificant)

In order to investigate impacts of overcrowded housing, we used a previously defined model of social overcrowding (SOC) in which mice are housed 10 per cage *vs*. 5 mice per cage that US animal welfare agencies view as normal housing (NH) for the cage size used in our vivarium (Figure 1a)^21^. We subjected SOC and NH mice to behavioral analysis following 10–12 weeks of such housing. SOC-housed mice traveled further, and did so at greater speed, in the elevated plus maze compared with NH mice. Moreover, SOC mice entered more often in the dark compartment of the light/dark box apparatus, without significant alterations in the open field test (**Figure S1**), indicating that, in our hands, the SOC model induced modest alterations in mice behavior without central impact on stress and anxiety-related behavior. However, these changes in behavior were accompanied by a more than twofold elevation in circulating levels of the adrenal gland-derived stress hormone corticosterone (NH: 26.40 mg/dL ± 8.31 mg/dL SOC: 82.90 mg/dL ± 19.84 mg/dL, *P* < .001, Figure 1b). Furthermore, SOC-housing impacted metabolism, as reflected by a trend toward an increased weight gain (Figure 1c) and a significant increase in fasting blood glucose level (NH: 80.25 mg/dL ± 1.11 mg/dL, SOC: 85.50 mg/dL ± 1.00 mg/dL, *P* = .04, Figure 1g). Collectively, these data comport with previous findings that SOC housing induces chronic stress that can detrimentally impact metabolism.

### SOC-induced stress associated with low-grade intestinal inflammation

**Figure 2.**
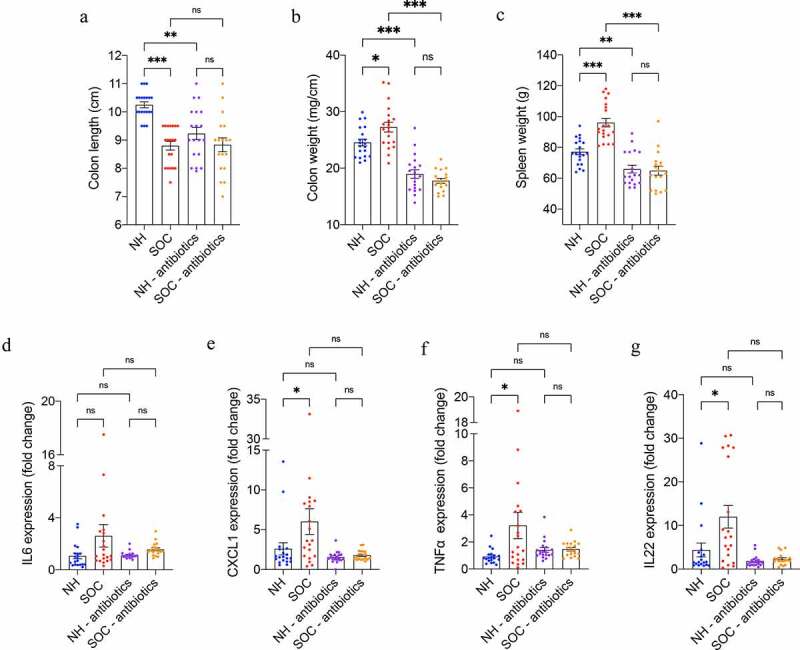
**SOC housing associates with low-grade intestinal inflammation in a microbiota-dependent manner**. Mice were subjected to SOC or normal housing for 19 weeks with or without antibiotic treatment. Colon length (a); colon weight (b) and spleen weight (c) after 19 weeks of SOC housing. Colonic mRNA levels of IL6 (d), CXCL1 (e), TNFα (f), and IL22 (g) were analyzed by qRT-PCR. Data are the means ± SEM, points represent individual mice (N = 20). Significance was determined using Kruskal-Wallis corrected for multiple comparisons with a Dunn’s test, or Brown-Forsythe and Welch ANOVA corrected for multiple comparisons with a Dunnett test, or one-way ANOVA corrected for multiple comparisons with a Bonferroni test (*p ≤ 0,05; **p ≤ 0,01; ***p ≤ 0,001; n.s. indicates nonsignificant)

Considering that psychological stress can promote intestinal inflammation and that low-grade gut inflammation can promote metabolic syndrome^22^, we next examined the extent to which SOC drove gut inflammation by measuring macroscopic and molecular indices of gut inflammation in NH- and SOC-housed mice. As presented in Figure 2a**-b**, SOC induced a significant decrease in colon length and increase in colon weight, thus indicating a state of chronic low-grade intestinal inflammation, while lack of a significant increase in a widely used fecal marker of inflammation, namely lipocalin-2 (**Figure S3a**) indicated lack of overt inflammation. Further demonstrating the development of chronic inflammation, SOC mice also developed mild splenomegaly (Figure 2c). To assay low-grade inflammation at the molecular level, colonic mRNA was extracted and subjected to q-RT-PCR for the quantification of inflammation-related cytokines. As presented Figure 2d**-g**, this approach revealed significant increases in CXCL1, TNF-α as well as IL22, further demonstrating that chronic low-grade intestinal inflammation had developed in SOC mice. The extent of elevated expression level of these inflammation-related cytokines did not correlate with corticosterone levels in SOC-housed mice (**Figure S2**).

### SOC promoted alterations in intestinal microbiota composition

**Figure 3.**
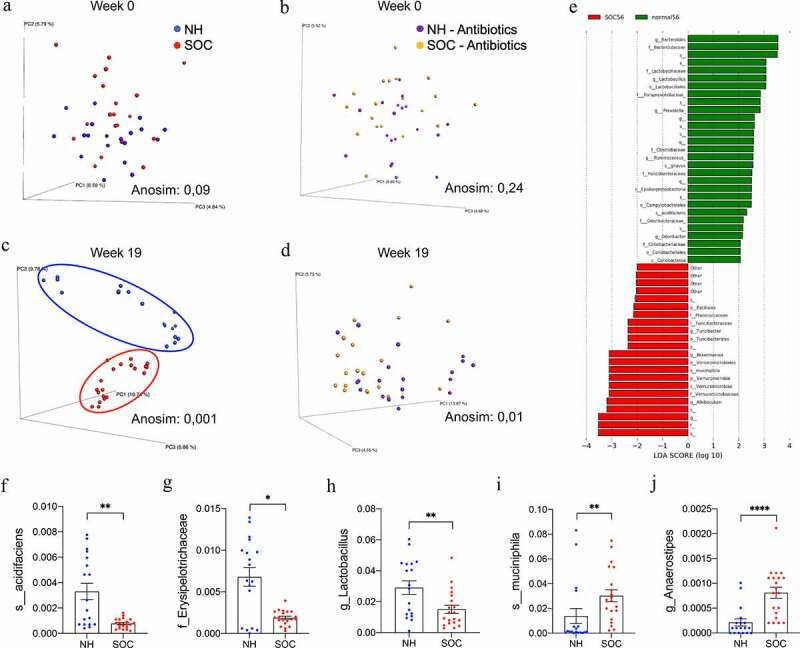
**SOC alters intestinal microbiota composition**. Mice were subjected to SOC or normal housing for 19 weeks with or without antibiotic treatment. Principal coordinates analysis of the unweighted UniFrac distance matrix of NH and SOC mice (a,c) and antibiotic-treated NH and SOC mice (b,d) at 0 and 19 weeks of treatment. LEfSe analysis for relative microbial abundance in SOC mice compared with NH mice at 19 weeks of treatment (e) and representation of select SOC-induced alteration of gut microbiota composition (f-j). Data are the means ± SEM, points represent individual mice (N = 20). Significance was determined using *t*-test or Anosim (*p ≤ 0,05; **p ≤ 0,01; ***p ≤ 0,001; n.s. indicates nonsignificant)

Several inducers of gut inflammation, including psychological stress, are reported to act by disturbing the relationship between the intestine and its commensal microbiota. Hence, we next measured the impact of SOC on fecal microbiota composition of NH- and SOC-housed mice through 16S rRNA sequencing. Prior to the initiation of the housing strategy, microbiota composition did not differ between NH and SOC mice (Figure 3a) but differed starkly following the housing intervention, as revealed by distinct clustering when using principal coordinate analysis of the unweighted Unifrac distance (Figure 3c, Anosim *P* = .001). Importantly, with four NH cages and two SOC cages being used, a cage clustering was observed but surpassed by the housing strategy (**Figure S3e**). The extent of SOC-induced microbiota composition alterations did not correlate with proinflammatory gene expression nor corticosterone levels (**Figure S3g-k**).

While microbiota alpha diversity (species richness), composition at the family level, as well as total bacterial load were not altered between groups (**Figure S3b-d**), linear discriminant analysis effect size (LEfSe), used to identify microbiota members significantly altered between groups^23^ revealed numerous taxa that differed in relative abundance between NH and SOC mice (Figure 3e). More specifically, SOC-housing induced a significant reduction in *Bacteroides acidifaciens* species and Erysipelotrichaceae family members, as well as intestinal health-associated *Lactobacillus* genus (figure 3f**-h**). Additionally, SOC-housing significantly increased abundance of *Akkermansia muciniphila* species and *Anaerostipes* genus (Figure 3i**-j**). Correlation analysis identified select microbiota member abundances that correlate positively or negatively with corticosterone levels and which, when used in combination, can efficiently predict corticosterone levels in SOC-housed mice (**Figure S4a-b**, R2 = 0.9988) but not in NH-housed mice (**Figure S4a, c**, R2 = 0.1402). Altogether, these data demonstrate that SOC induces profound alterations in intestinal microbiota composition that associate with stress, low-grade intestinal inflammation, and altered metabolism.

*SOC-induced stress, low-grade intestinal inflammation and altered metabolism displayed microbiota dependence*

SOC-induced alterations in microbiota composition could potentially be a cause and/or consequence of stress, intestinal inflammation, and metabolic changes. Hence, as an initial step toward investigating the role played by intestinal microbiota in these events, we ablated bacteria *via* broad-spectrum antibiotics (ampicillin 1% + neomycin 1%) applied to both groups throughout the experiment. This approach eliminated 99.99% of the intestinal microbiota, as demonstrated by total bacterial load quantification through q-PCR approach (**Figure S3d**). Moreover, the species composition in the remaining bacteria did not differ between NH and SOC mice (Figure 3b-d **and S3f**). Strikingly, such microbiota depletion eliminated all the above-described differences between NH and SOC mice. More specifically, when compared under antibiotic treatment, NH and SOC did not differ in circulating corticosterone levels (Figure 1b) nor fasting glucose (Figure 1g), while antibiotic per se wasn’t sufficient to alter these parameters in NH mice. Moreover, previously observed SOC-induced alteration in colon length, spleen weight, and intestinal cytokine expression were abrogated following microbiota depletion (Figure 2). Furthermore, antibiotic-mediated abrogation of SOC-associated phenotypes correlated with decreased levels of microbiota taxa that associated with SOC housing and/or circulating corticosterone levels (**Figure S4d-e**). Hence, these results accord with the central role played by the intestinal microbiota in mediating impacts of SOC on stress and inflammation.

### Transplant of SOC microbiotas to NH mice promotes gut inflammation and hyperglycemia

**Figure 4.**
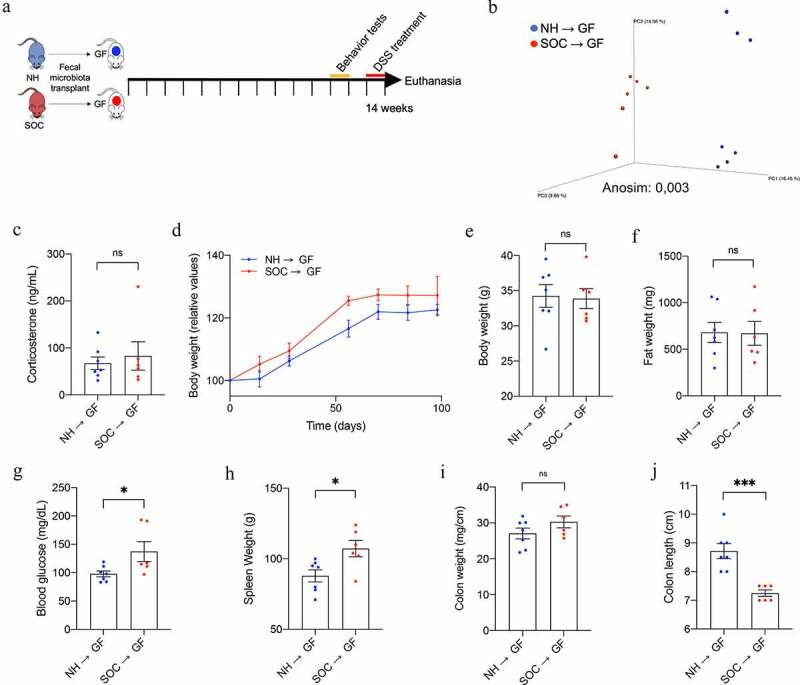
**The intestinal microbiota from SOC-housed mice is sufficient to impact intestinal homeostasis when transplanted to normally housed germfree mice**. 4-week-old germ-free mice were transplanted with microbiota from SOC- or NH-(housed) mice, as presented in (a). Principal coordinates analysis of the unweighted UniFrac distance matrix of NH → GF and SOC → GF microbiota 14 weeks after transplantation (b). Corticosterone serum levels were analyzed by ELISA (c). Body weight over time (d); final body weight (e); epididymal white adipose tissue weight (f). 15-h fasting blood glucose level 13 weeks after transplantation (g); spleen weight (h); colon weight (mg/cm) (i) and colon length (j). Data are the means ± SEM, points represent individual mice (N = 6–7). Significance was determined using *t*-test or Anosim (*p ≤ 0,05; ***p ≤ 0,001; n.s. indicates nonsignificant)

We next sought to investigate the extent to which SOC-associated alterations in microbiota might be sufficient to drive other consequences of SOC. We performed fecal microbiota transplantation from NH- and SOC-housed mice to normally housed germfree mice (Figure 4a **and S5**). For this purpose, fecal suspension from NH- and SOC-housed mice were transferred by oral gavage to germfree C57BL/6 mice (4-week-old, N = 6–7) that were subsequently housed in Isocages (Techniplast, West Chester, Pennsylvania, USA), which prevents contamination from environmental microbes^24^. Fourteen weeks post-transplant, mice were administered low-dose DSS as a means of investigating their proneness to intestinal inflammation. This approach led to clear differences in fecal microbiota composition in NH → GF *versus* SOC → GF mice, as indicated by principal coordinate analysis (Figure 4b), indicating that at least some of the differences that resulted from SOC were maintained even when mice were housed at normal density. Transfer of SOC microbiota did not impact circulating corticosterone levels (Figure 4c) or behavior (**Figure S5d-k**), but was sufficient to transfer alterations in metabolism (body weight and 15-h fasting glucose levels, Figure 4d**,g**) as well as some markers of low-grade intestinal inflammation, including splenomegaly and colon shortening (Figure 4h**,j and S6**). Together, these data demonstrate that alterations in microbiota resulting from SOC housing are not purely a consequence of inflammation and altered metabolism but, rather, contribute to SOC-induced phenotypes.

## Discussion

Gut microbiota has long been recognized to be germane to pathogenesis of Inflammatory bowel diseases. More recently, microbiota has been appreciated to be an important determinant of diseases characterized by low-grade inflammation including obesity and diabetes. These diseases are not strictly caused by specific microbial taxa per se but rather are influenced by the intersection of complex interactions between genetics, environment, and gut microbiota^25^. The central conclusion of this study is that this concept applies to the array of disorders, including behavioral abnormalities and stress-induced metabolic disorders that are associated with social overcrowding. Briefly, we, herein used a mouse model of social overcrowding (SOC) to investigate the interrelationship between chronic stress, intestinal microbiota, gut inflammation, and consequences associated with this state. We found that chronic stress induced by SOC housing was sufficient to increase circulating levels of the stress-related hormone corticosterone, which associated with dysglycemia and chronic low-grade intestinal inflammation. SOC also led to profound alterations in the composition of the gut microbiota composition, which was critical for SOC’s detrimental consequences.

While SOC’s impact on gut microbiota has not been previously reported, our observations that SOC induces metabolic dysregulation is consistent with published work of Lin and colleagues who reported that SOC increased serum corticosterone and adiposity^21^.Although we suspect that some of the phenotypic differences between this study and the work herein, particularly the duration of the impacts, may reflect differences in the protocol length and overcrowded housing density (8 mice in 480,5 m^2^ for 9 weeks in *Lin et al. 2016*^21^ study, *versus* 10 mice in 484 cm^2^ for 19 weeks in the present study). Lower density in *Lin et al. 2016* study might indeed allow chronic stressor habituation, whereas our protocol enables conservation of the chronic stress induced by SOC-housing, efficiently maintaining corticosterone increased serum levels for 19 weeks in SOC mice. Another possibility which will require future investigation is, in light of the central role played by the intestinal microbiota in driving SOC-induced metabolic and inflammatory phenotypes, the possibility that different microbiota composition from independent housing facilities could dramatically impact the outcome of the SOC procedure.

While understanding how altered microbiota might interact with the intestine in the context of SOC will require further investigation, potential mechanisms can be envisaged. Chronic stress exposure is known to alter small and large intestine physiology through, for example, disruption of iron secretion and intestinal epithelium permeability^26^. Such modification of the mucosal environment could be a mechanism involved in the building of SOC-associated phenotypes in a way that could also explain their dependency toward microbiota alterations. Moreover, exacerbated intestinal inflammation is likely to modify communication through the gut-brain axis. Intestinal microbiota is known to modulate the host stress response through an array of mechanisms, including changes in pro-inflammatory cytokine level in the hippocampus which is known to modulate stress^16^. and expression of genes involved in stress response^27^. Hence, we submit that future studies are needed to investigate microbiota-dependent alteration of corticosterone level observed in SOC.

Importantly, SOC-induced microbiota alteration did not influence microbiota taxonomic richness nor alter its composition at the family levels but, rather, impacted abundance of key microbiota members such that relative abundance of select species was sufficient to predict corticosterone levels. Microbiota members exhibiting decreased abundance in response to SOC housing included *Bacteroides acidifaciens* and *Lactobacillus*^28^. *Lactobacillus* depletion following chronic stress has been previously described^18^, and therapeutic restoration of *Lactobacillus* improved metabolic homeostasis and corrected stress-induced behaviors alteration as well as corticosterone levels normalization in both mice^19^ and human^29,30^. *Lactobacillus* is also associated with the reduction of intestinal inflammation, including reduction of proinflammatory cytokine levels and dampening of intestinal immune cells infiltration^30,31^. Hence, *Lactobacillus* – and other microbiota members – may have capacity to impact stress and intestinal inflammation, suggesting that these microbiota members could be central in driving SOC associated phenotypes. Further supporting such hypothesis, our use of fecal microbiota transplantation of NH and SOC microbiota to germfree-mice demonstrated that alterations in microbiota resulting from SOC housing are not purely a consequence of inflammation and altered metabolism but, rather, contribute to these SOC-induced phenotypes. However, in these recipient mice, we did not observed alterations in corticosterone serum level and inflammatory cytokines, and alterations in *Lactobacillus* abundance wasn’t transferred (data not shown), suggesting that (i) select microbiota members may not be efficiently transferred through FMT and/or (ii) that SOC-induced stress is required to sustain their levels in the intestine. Alternatively, our inability to fully restore SOC-associated intestinal inflammation, behavior alteration and decreased increased corticosterone level through FMT suggests that, while SOC-induced microbiota alterations are required, they are likely not sufficient to drive the full consequences of SOC housing. Hence, future mechanistic studies appear warranted in order to investigate physiological impacts of microbiota members found to associate with SOC housing.

In conclusion, our study suggests that overcrowded housing is sufficient to induce chronic stress that associates with intestinal inflammation and metabolism deregulations, with a central role played by the intestinal microbiota. This supports the importance of considering interaction between environmental factors and gut microbiota in the onset of chronic inflammatory diseases. Moreover, it suggests that, in addition to other microbiota stressors such as ultra-processed food consumption^32^, increased density housing is part of the chronic stressors that contribute to the disproportionate impact of chronic inflammatory diseases amongst individuals of low socio-economic status^3,33–35^. Finally, while our data demonstrate that SOC-associated microbiota contribute to SOC-induced phenotypes, future studies appear needed in order to identify microbiota members involved and to characterize the impact of these members at steady state as well as in other inflammatory models.

## Methods

### Mice

Four weeks old male C57BL/6 mice were purchased from Jackson Laboratories. Mice were housed in groups of 5 (control, 8 cages) or 10 (social overcrowding, 4 cages) for 19 weeks in Animal Care Systems Optimice ® cages (484 cm^2^, designed to house 5 mice). All mice were kept under a 12-h light/dark cycle and had free access to standard chow diet and water (water-treated group, four cages of normally housed, and two caged of social overcrowding) or water with antibiotics (ampicillin, neomycin, 1%, Sigma, antibiotic-treated group, four cages of normally-housed, and two caged of social overcrowding). Cages from all groups were changed biweekly. Body weight were measured and feces were collected every other week. All procedures were approved by the Animal Care and Use Committee of Georgia State University (protocol #A18006). After 19 weeks, mice were fasted for 5 h and serum was collected. Mice were then euthanized and colon length, colon weight, spleen weight, and adipose weight were measured. Organs were collected for downstream analysis.

### Behavioral testing

#### Open field test

The open field consisted of an empty square arena (60×60×40 cm) constructed of plywood and painted white. The bottom of the box was divided into 25 equal squares on average, with 9 squares in the center being the central area. Mice were placed individually in the corner of the open field apparatus, then the spontaneous activities were recorded for 10 min using a video-tracking program. The total distance and speed were analyzed as measures of spontaneous activity. The time spent in the center was measured as an index of anxiety-like behavior.

#### Elevated plus maze

The EPM was used to measure anxiety-related behavior. The EPM consists of two opposite open-arms (50×10 cm) and two opposite closed-arms (50×10 cm) with 30 cm opaque high walls, elevated 50 cm above the ground. Arm entry defined as center point of the mouse entering an arm. Each mouse was gently placed in the center of the maze and explored for 5 min. The total distance and speed were analyzed as measures of spontaneous activity. The amounts of time spent in the center, open and closed arms were recorded and quantified by AnyMaze, and used as an index of anxiety-like behavior.

#### Light/dark box test

The L/D box test consists of a chamber divided into a light and dark compartment with an insert connecting the compartments. Mice were placed in the light compartment facing away from the entry into the dark chamber and allowed to freely investigate the chamber for 5 min. The amounts of time spent in the light and dark compartment, and the number of entries into the dark chamber were quantified by AnyMaze and used as an index of anxiety-like behavior.

### Quantification of serum corticosterone by ELISA

Serum corticosterone levels were determined using Corticosterone Parameter Assay (R&D Systems). Briefly, corticosterone present in supernatant competed with a fixed amount of horseradish peroxidase (HRP)-labeled corticosterone for sites on a sheep polyclonal antibody specific for corticosterone. A standard curve was used in order to determine the serum corticosterone concentration.

### Overnight fasting blood glucose measurement

Mice were placed in a clean cage and fasted for 15 h. Blood glucose concentration was then determined using a Nova Max Plus Glucose Meter and expressed in mg.dl.^−1^

### Food intake measurement

Groups of mice were placed in a clean cage with a known amount of food. Twenty-four hours later, the amount of remaining food was measured with the difference viewed as food intake per 24 h.

### Colonic RNAs extraction and q-RT-PCR analysis

Distal colon was collected during euthanasia and placed in RNAlater. Total mRNAs were isolated from colonic tissues using TRIzol (Invitrogen, Carlsbad, CA) according to the manufacturer’s instructions and as previously described^36^. For mRNAs extracted from DSS-treated mice, an additional Lithium Chloride purification was performed, as previously described. Quantitative RT-PCR were performed using the Qiagen kit QuantiFast® SYBR® Green RT-PCR in a LigthCycler® 480 instrument (Roche Molecular Systems, Inc) with specific mouse oligonucleotides (**Table S1**). Gene expression are presented as relative values using the Ct approach with 36B4 housekeeping gene.

### Antibiotic treatment

Mice were placed on broad-spectrum antibiotics ampicillin (1.0g/L) and neomycin (0.5 g/L) in drinking water for 14 weeks.

### Quantification of fecal bacterial load

Total bacterial DNA was isolated from feces using QIAamp DNA Stool Mini Kit (Qiagen). DNA was then subjected to quantitative PCR using universal 16S rRNA primers 515 F: 5ʹ GTGCCAGCMGCCGCGGTAA-3ʹ and 806 R: 5ʹ-GGACTACHVGGGTWTCTAAT-3ʹ to measure total bacterial load.

### Microbiota analysis by 16 S rRNA gene sequencing

16S rRNA gene amplification and sequencing were done using the Illumina MiSeq technology following the protocol of Earth Microbiome Project with their modifications to the MOBIO PowerSoil DNA Isolation Kit procedure for extracting DNA (www.earthmicrobiome. org/emp-standard-protocols). Bulk DNA were extracted from frozen extruded feces using a PowerSoil-htp kit from MoBio Laboratories (Carlsbad, California, USA) with mechanical disruption (bead-beating). The 16 S rRNA genes, region V4, were PCR amplified from each sample using a composite forward primer and a reverse primer containing a unique 12-base barcode, designed using the Golay error-correcting scheme, which was used to tag PCR products from respective samples^37^. We used the forward primer 515 F 5ʹ- *AATGATACGGCGACCACCGAGATCTACACGCT*XXXXXXXXXXXX**TATGGTAATT*GT***GTGYCAGCMGCCGCGGTAA-3ʹ: the italicized sequence is the 5ʹ Illumina adapter, the 12× sequence is the Golay barcode, the bold sequence is the primer pad, the italicized and bold sequence is the primer linker and the underlined sequence is the conserved bacterial primer 515 F. The reverse primer 806 R used was 5ʹ-*CAAGCAGAAGACGGCATACGAGAT***AGTCAGCCAG*CC*** GGACTACNVGGGTWTCTAAT-3ʹ: the italicized sequence is the 3ʹ reverse complement sequence of Illumina adapter, the bold sequence is the primer pad, the italicized and bold sequence is the primer linker and the underlined sequence is the conserved bacterial primer 806 R. PCR reactions consisted of Hot Master PCR mix (Five Prime), 0.2 μM of each primer, 10–100 ng template, and reaction conditions were 3 min at 95°C, followed by 30 cycles of 45 s at 95°C, 60 s at 50°C, and 90 s at 72°C on a Biorad thermocycler. Four independent PCRs were performed for each sample, combined, purified with Ampure magnetic purification beads (Agencourt), and products were visualized by gel electrophoresis. Products were then quantified (BIOTEK Fluorescence Spectrophotometer) using Quant-iT PicoGreen dsDNA assay. A master DNA pool was generated from the purified products in equimolar ratios. The pooled products were quantified using Quant-iT PicoGreen dsDNA assay and then sequenced using an Illumina MiSeq sequencer (paired-end reads, 2 × 250 bp) at Cornell University, Ithaca.

### 16S rRNA gene sequence analysis

Forward and reverse Illumina reads were joined using the fastq-join method^38,39^, sequences were demultiplexed, quality filtered using Quantitative Insights Into Microbial Ecology (QIIME, version 1.8.0) software package^39^. QIIME default parameters were used for quality filtering (reads truncated at first low-quality base and excluded if: (1) there were more than three consecutive low-quality base calls (2), < 75% of read length was consecutive high-quality base calls (3), at least one uncalled base was present (4), > 1.5 errors were present in the barcode (5), any Phred qualities were below 20, or (6) the length was < 75 bases). Sequences were clustered to operational taxonomic units (OTUs) using UCLUST algorithm^40^ with a 97% threshold of pairwise identity (without the creation of new clusters with sequences that do not match the reference sequences), and taxonomically classified using the Greengenes reference database 13_8. A single representative sequence for each OTU was aligned and a phylogenetic tree was built using FastTree^41^. The phylogenetic tree was used for computing the weighted and unweighted UniFrac distances between samples^42,43^, rarefaction were performed and used to compare abundances of OTUs across samples. Principal coordinates analysis plots were used to assess the variation between experimental groups (beta diversity). Alpha diversity curves were determined for all samples using the chao1 index. LEfSe (LDA Effect Size) was used to investigate bacterial members that drive differences between groups^23^. Unprocessed 16S sequencing data are deposited in the European Nucleotide Archive under accession numbers PRJEB44512.

### Quantification of fecal LCN2 by ELISA

For quantification of fecal LCN2 by ELISA, frozen fecal samples were reconstituted in PBS containing 0.1%Tween 20 to a final concentration of 100 mg.ml^−1^ and vortexed for 20 min to produce a homogenous fecal suspension^36^. These samples were then centrifuged for 10 min at 14,000 g and 4°C. Clear supernatants were collected and stored at −20°C until analysis. LCN2 levels were estimated in the supernatants using Duoset murine LCN2 ELISA kit (R&D Systems, Minneapolis, Minnesota) using the colorimetric peroxidase substrate tetramethylbenzidine, and optical density was read at 450 nm (VersaMax microplate reader).

### Microbiota transplantation

Fecal contents from NH and SOC mice were suspended in 30% glycerol diluted in PBS (1.0 ml) and stocked at −80°C until analysis. Germfree C57BL/6 mice (4 week-old) were removed from a ParkBio isolator and were orally administered 200 μl of fecal suspension made using glycerol stocks. Recipient mice were transplanted using homogenized fecal samples pooled from 2 NH and 2 SOC mice. Transplanted mice were then housed in isolated ventilated cages, Isocages (Techniplast, West Chester, Pennsylvania, USA) for 14 weeks and fed autoclaved Purina Rodent Chow # 5021^24,44,45^. On week 12, behavioral testing was performed (open field test, elevated plus maze and light-dark box). On week 14, mice were treated with dextran sulfate sodium (DSS, 1%) for 7 days prior euthanasia and tissue collection. For each condition (NH → GF and SOC → GF), two cages containing 3–4 animals were used, and transplanted mice were then monitored as previously described.

### Statistical analysis

Significance was determined using t-test or one-way group analysis of variance (ANOVA) with Bonferroni’s multiple comparisons test (GraphPad Prism software, V.8). Significance of data that did not respect normality and homoscedasticity postulates was tested using Kruskal-Wallis corrected for multiple comparisons with a Dunn’s test and Brown-Forsythe and Welch ANOVA corrected for multiple comparisons with a Dunnett test, respectively. For significance of body weight over time, a two-way ANOVA corrected for multiple comparisons with a Bonferroni test was used. Differences were noted as significant *p ≤ 0,05; ** p ≤ 0,01; *** p ≤ 0,001; **** p ≤ 0,0001; n.s. indicates nonsignificant. For clustering analysis on principal coordinate plots, categories were compared, and statistical significance of clustering was determined via the analysis of similarities (Anosim).

## Supplementary Material

Supplemental Material

## Abbreviations:

Lcn-2, lipocalin-2; LEfSe, LDA Effect Size.
