# Supplementary material for: Social overcrowding impacts gut microbiota, promoting stress, inflammation, and dysglycemia
Source: Gut Microbes. 2021 Dec 2;13(1):2000275. doi: 10.1080/19490976.2021.2000275 (PMC8726700; doi:10.1080/19490976.2021.2000275)
Supplement: Supplemental Material [file KGMI_A_2000275_SM6531.zip › Supplementary information/Supplemental material.docx]

**Supplemental figure legends**

**Figure S1: SOC housing affects mice behavior in a microbiota dependent manner.** Mice were subjected to SOC or normal housing for 19 weeks with or without antibiotic treatment. Behavior was analyzed between week 10 and week 12. Percent of the total time spent in the center and side areas of the open field test (**A**), total distance and speed of NH, SOC and antibiotic treated mice in the open field test (**B-C**). Percent of the total time spent in the open arms, closed arms and center of the elevated plus maze (**D**), total distance and speed of NH, SOC and antibiotic treated mice in the elevated plus maze (**E-F**). Percent of the total time spent in the light area and dark area of the light/dark box (test) (**G**), and number of entries in the dark area (**H**) of the light/dark box (test). Data are the means +/- SEM, points represent individual mice (N=20). Signiﬁcance was determined using one-way ANOVA corrected for multiple comparisons with a Bonferroni test (*p≤ 0,05 ; **p≤ 0,01; n.s. indicates non-signiﬁcant).

**Figure S2: Corticosterone level was not associated with proinflammatory cytokine expression in SOC-housed mice.** Mice were subjected to SOC or normal housing for 19 weeks. Colonic mRNA levels of pro-inflammatory cytokine are presented for NH mice, SOC mice harboring intermediate corticosterone level (<100ng/mL) and SOC mice harboring high corticosterone level (>100ng/mL). Signiﬁcance was determined using Kruskall-Wallis corrected for multiple comparisons with a Dunn’s test (n.s. indicates non-signiﬁcant).

**Figure S3: SOC housing impacted gut microbiota composition but not species richness nor lipocalin-2 fecal levels.** Mice were subjected to SOC or normal housing for 19 weeks with or without antibiotic treatment. Fecal levels of the inflammatory marker lipocalin-2 levels at day 0, 56 and 112 (**A**). Alpha diversity (**B**), and taxonomic visualization of the intestinal microbiota at the family level (**C**) after 19 weeks of SOC housing. Fecal bacterial load after 19 weeks of antibiotic treatment (**D**). Principal coordinates analysis of the unweighted UniFrac distance matrix of NH and SOC mice identified by cages (**E**) and antibiotic-treated NH and SOC mice identified by cages (**F**) at 19 weeks of treatment. Correlation analysis between the Unifrac distances mean between each mice and NH mice and corticosterone level (**G**) and pro-inflammatory cytokine mRNA (**H, I, J, K**). Data are the means +/- SEM, points represent individual mice (N=20). Signiﬁcance was determined using t-test (****p≤ 0,0001 ; n.s. indicates non-signiﬁcant).

**Figure S4: Abundance of select OTUs can predict corticosterone levels in SOC mice.** Mice were subjected to SOC or normal housing for 19 weeks with or without antibiotic treatment. OTUs with the highest (9) and the lowest (9) correlation coefficient calculated between corticosterone levels and OTUs abundance were selected in SOC mice and are listed in (**A**). Table presents OTUs corresponding taxonomy, correlation coefficient calculated between corticosterone levels and OTUs abundance in SOC mice, and p-values of t-tests assessing OTUs different abundance between NH and SOC mice. Multiple linear regression of 18 OTUs to predict corticosterone level in SOC mice (**B**) and in NH mice **(C**). OTU 338644 and 178735 abundance in NH and SOC mice with or without antibiotic treatment (**D, E**). Data are the means +/- SEM, points represent individual mice (N=20). Signiﬁcance was determined using one-way ANOVA corrected for multiple comparisons with a Bonferroni test (***p≤ 0,001; n.s. indicates non-signiﬁcant).

**Figure S5:** **SOC microbiota transplantation impacts on behavior.** 4-week old germ-free mice were transplanted with microbiota from SOC- or NH-housed mice. Behavioral testing was performed 12 weeks after the transplantation. Alpha diversity (**A**), taxonomic visualization of the intestinal microbiota at the family level (**B**) and fecal levels of the inflammatory marker lipocalin-2 (**C**) were analyzed 14 weeks after transplantation. Percent of the total time spent in the center and side areas of the open field test (**D**), total distance and speed of NH, SOC and antibiotic treated mice in the open field test (**E-F**). Percent of the total time spent in the open arms, closed arms and center of the elevated plus maze (**G**), total distance and speed of NH, SOC and antibiotic treated mice in the elevated plus maze (**H-I**). Percent of the total time spent in the light area and dark area of the light/dark box (test) (**J**), and number of entries in the dark area (**K**) of the light/dark box (test). Data are the means +/- SEM, points represent individual mice (N=6-7). Signiﬁcance was determined using t-test (n.s. indicates non-signiﬁcant).

**Figure S6:** **SOC microbiota transplantation impacts on colonic expression of molecular markers of inflammation.** 4-week old germ-free mice were transplanted with microbiota from SOC- or NH-housed mice. Colonic mRNA levels of IL6 (**A**), CXCL1 (**B**), IL22 (**C**), and TNFα (**D**) were analyzed by qRT-PCR. Data are the means +/- SEM, points represent individual mice (N=6-7). Signiﬁcance was determined using t-test (*p≤ 0,05; n.s. indicates non-signiﬁcant).

**SUPPLEMENTAL TABLE**

**Table S1.** Oligonucleotides used. Related to Figure 2 and S5.

| **Oligonucleotides name** | **Oligonucleotide sequence (5’-3’)** |
| --- | --- |
| *IL6*-F  *IL6*-R | ACAAGTCGGAGGCTTAATTACACAT  TTGCCATTGCACAACTCTTTTC |
|  |  |
| *36B4*-F  *36B4*-R | TCCAGGCTTTGGGCATCA  CTTTATTCAGCTGCACATCACTCAGA |
|  |  |
| *CXCL1*-F  *CXCL1*-R | TTGTGCGAAAAGAAGTGCAG  TACAAACACAGCCTCCCACA |
|  |  |
| *IL22*-F  *IL22*-R | GTCAACCGCACCTTTATGCT  GTTGAGCACCTGCTTCATCA |
|  |  |
| *TNF-α*-F  *TNF-α*-R | AGGCTGCCCCGACTACGT  GACTTTCTCCTGGTATGAGATAGCAAA |
|  |  |
